# Development and Validation of a Tuberculosis Medication Adherence Scale

**DOI:** 10.1371/journal.pone.0050328

**Published:** 2012-12-12

**Authors:** Xiaoxv Yin, Xiaochen Tu, Yeqing Tong, Rui Yang, Yunxia Wang, Shiyi Cao, Hong Fan, Feng Wang, Yanhong Gong, Ping Yin, Zuxun Lu

**Affiliations:** 1 School of Public Health, Tongji Medical College, Huazhong University of Science and Technology, Wuhan, China; 2 Hubei Center for Disease Control and Prevention, Wuhan, China; 3 Department of Social Medicine and Education, School of Public Health, Nanjing Medical University, Nanjing, China; 4 School of Public Health and Primary Care, The Chinese University of Hong Kong, Hong Kong; 5 Department of Epidemiology and Biostatistics, School of Public Health, Tongji Medical College, Huazhong University of Science and Technology, Wuhan, China; University of Otago, New Zealand

## Abstract

**Background:**

Medication adherence is critical in Tuberculosis (TB) treatment success, but existing tools are inadequate in identifying non-adherents, reasons for non-adherence or interventions to improve adherence. This study intended to fill the gap by developing and validating a TB medication adherence scale (TBMAS).

**Methods:**

An initial 41-item TBMAS was designed through review of literature, consultation from an 8-member clinical expert panel and a 15-patient focus group, and pilot-testing in 25 TB patients. The questionnaire was validated in 438 patients who visited 23 community health centers for TB treatment in Wuhan from September 1, 2010, to August 31, 2011, using pharmacy refill records in a 15-week period as external criteria for medication adherence. After removing redundant and cross-loading items, the internal consistency, reliability and validity of TBMAS in identifying non-adherents were examined.

**Results:**

The final TBMAS included 30 items scored on a 5-point Likert scale, and these items were loaded in nine distinct factors that explained 65% of cumulative variance among respondents. Cronbach's alpha, test-retest reliability and split-half reliability were 0.87, 0.83, and 0.85, respectively. Convergent validity was supported by statistically significant associations between TBMAS scores and adherence measured by pharmacy refill records. Receiver Operating Characteristics curve analysis suggested a cut-off point at 113, with which TBMAS showed a positive predictive value of 65.5% and sensitivity of 82.9% in identifying non-adherents.

**Conclusion:**

TBMAS demonstrated satisfactory internal consistency, reliability and validity in identifying TB patients with poor adherence and potential causes for non-adherence.

## Introduction

Since 1991, Directly Observed Therapy, Short-course (DOTS), has been the primary intervention strategy for Tuberculosis (TB) control worldwide, promoted vigorously by the World Health Organization (WHO) and implemented by almost all WHO member countries [1]. This strategy has four key technical pillars: (1) detection of smear-positive pulmonary TB using sputum microscopy, in patients presenting themselves to public clinics; (2) directly observed treatment with short-course chemotherapy; (3) guaranteed continuous drug therapy; and (4) a case reporting system tracking treatment outcomes [2]. Central to this strategy is the second pillar, directly observed treatment (DOT), which recognizes the pivotal role of patient adherence in TB treatment and the importance of close supervision by medical professionals in assuring patient adherence. Despite its theoretical appeal and overall success, DOTS is not without controversies. First, DOT can be too costly and impractical for some patients, especially in developing countries [3]–[5]. Second, there is limited evidence that DOT is better than other strategies in assuring patient adherence; for example, some studies in China showed no significant difference between DOT group and self-supervision group in the rates of TB treatment completion [6], [7]. Third, there is a concern that DOT shifts the responsibility for treatment adherence from TB patients to medical professionals, which can take away patients' sense of self-control and diminish their motivation for self-care, resulting in poor adherence [5]. In light of these controversies, TB control communities have explored various alternative strategies to promote adherence, emphasizing the active involvement of TB patients in the treatment process and making measuring and improving patient adherence a prominent issue in TB control efforts [8]–[11].

There are many ways to assess patient adherence, such as pharmacy record review and pill counts, but they are not feasible for routine clinical practice because they require excessive physician or patient time and resources [12]. The most common approach to medication adherence assessment is patient self-reports collected through face-to-face interview, telephone interview or self-administration of questionnaires, such as the Morisky, Green and Levine adherence scale (MGLS), the Brief Medication Questionnaire (BMQ) and the Medication Adherence Rating Scale (MARS) [13]–[15]. These questionnaires ask patients straightforward questions regarding adherence. For example, MGLS contains 4 items to be answered with “yes” or “no”: (1) Do you ever forget to take your medicine? (2) Are you careless at times about taking your medicine? (3) When you feel better, do you sometimes stop taking your medicine? And (4) Sometimes if you feel worse when you take the medicine, do you stop taking it? A person is considered to be non-adherent if he or she responds affirmatively to at least one question [13]. These available tools can be used to identify non-adherents, however their reliability and validity are controversial [16], and they offer little insight into the reasons for non-adherence or specific barriers to adherence, therefore, they are of limited value in developing targeted interventions to improve medication adherence [12]. Furthermore, these instruments are generic, none of them specifically designed or suited for assessing TB medication adherence.

This study was aimed at developing and validating a TB medication adherence scale (TBMAS) that incorporated the latest research both in general medication adherence, as reviewed above, and in TB specific medication adherence, where predictors for adherence such as patient behavior and patient-provider interaction in TB treatment have been explored [17], [18]. The resulting tool will help TB medical professionals identify not only TB patients with poor adherence but also potential reasons for non-adherence, and help them to design and implement targeted interventions to improve adherence.

## Methods

### Ethics statement

The study protocol and the questionnaire were reviewed and approved by the ethic committee of Huazhong University of Science and Technology. Participation was voluntary and all participants gave written informed consent before being involved in the study.

### Development of TBMAS

TBMAS was developed in four steps. In Step 1, we conducted comprehensive reviews of literature on patient adherence, identifying factors and potential self-report questions. In Step 2, we organized an advisory panel consisted of 8 physicians and nurse professionals to discuss the literature and relevant factors in TB medication adherence in the context of their clinical experiences. At the end of this step, we identified nine factors conceptually associated with medication adherence in TB patients: (1) communication with healthcare providers, (2) personal traits, (3) confidence in curing TB, (4) social support, (5) mood disorders, (6) lifestyle and habits, (7) coping style, (8) access to healthcare, and (9) forgetfulness. From these nine factors, 35 items or self-report questions were created to form the preliminary TBMAS. In step 3, we organized a patient focus group with 15 TB patients randomly selected from the TB Patient Registry in Wuhan, Hubei, which record personal, diagnostic and treatment information for all TB patients who has come into contact with the healthcare facilities in the city. These 15 TB patients were interviewed with the 35-item, semi-structured questionnaire. During the interviews, the patients were asked to complete the questionnaire and provide their points of view over each item, the entire survey, and any additional factors which they thought would affect adherence. Based on the focus group input, the questionnaire was refined and expanded to 41 items, with each item scored on a 5-point Likert scale ranging from “strongly disagree” to “strongly agree” and coded with values from 1 to 5.

In Step 4, the questionnaire was pilot-tested in 25 patients selected from the same TB registry to ensure that the questions were clear and understandable to all participants. The acceptability of the survey and the time required to complete it were also examined.

### Validation of TBMAS

The validation study of the 41-item TBMAS was conducted from September 1, 2010, to August 31, 2011, in 23 Community Health Centers (CHCs) in Wuhan, Hubei province. Patients were eligible if they were active TB patients confirmed by positive sputum assay or by X-ray, and if they had been under treatment for at least two months by the time of the recruitment. During the study period, 467 active TB patients were eligible, and 29 (6.21%) of them refused to participate; patient characteristics such as gender, age, education, marital status and income between patients who participated and who refused to participate were statistically insignificant.

The 438 participating patients were interviewed face-to-face using TBMAS by general practitioners (GPs) at the moment they visited the CHCs for a refill of TB medication. In order to assess test-retest reliability, 50 of the 438 patients were randomly sampled to complete TBMAS again 20 days later.

To establish external criteria for validating TBMAS, the pharmacy records of the 438 participating patients in a 15-week period were reviewed and the Continuous Multiple-Interval Medication Gaps (CMG) was calculated. In the city of Wuhan where this research was conducted, TB patients were provided with adequate medications only for a week at each visit, and they were required to visit CHCs for medication refills every week. The GPs were responsible for recording the time when patients should refill medications and when patients actually refilled medications. Since the entire TB treatment course lasted about six months, each patient is expected to make 25 refills. We reviewed each patient's refill records from the sixth refill for 15 refills to capture the patient's refill adherence in the mid-course of his treatment. CMG is a well-established objective measure of medication adherence based on pharmacy record, and is calculated by dividing the total number of days without medications (i.e. treatment gaps) between the first and last pharmacy fill by the number of days in this time period; the resulting CMG has a value ranging from 0 to1, and, in general, patients with CMG<0.2 is considered non-adherents [19], [20].

Once the three databases were compiled, we conducted the following analyses to screen redundant or non-informative items and estimate the reliability and validity of the resulting TBMAS.

#### Discrimination coefficient

The scores of the 438 patients responded to the 41-item TBMAS were sorted and the highest 25% and lowest 25% of respondents were retained. An item's discrimination coefficient was determined by the difference between the average scores of the item in these two groups; the greater the difference, the higher the discrimination power of the item. Any item with the discrimination coefficient <0.5 were considered insufficient in discrimination power and was therefore removed from the questionnaire.

#### Spearman correlation

Spearman correlation between each item and medication adherence measured by CMG was calculated to determine the overall relevance of the item in identifying adherence. Any item with insignificant correlation (p>0.05) was removed from the questionnaire.

#### Exploratory factor analysis

this analysis was performed to investigate the factor structure of the questionnaire. The number of inherent factors was determined using Catells's scree plot analysis, and the resulting factors were subjected to Varimax rotation. Items failing to show salient loadings on any of the factors and items with substantial cross-loadings on two or more factors were removed.

#### Cronbach's alpha

this statistic was calculated to measure the internal consistency of TBMAS.

#### Test-retest reliability

it was assessed by intraclass correlation coefficient (ICC) between the scores of the 50 patients who completed TBMAS twice.

#### Split-half reliability

it was estimated by Pearson correlation between the scores of the odd items and even items of TBMAS.

#### Construct validity

Confirmatory Factor Analysis using Varimax rotation was conducted to examine the underlying factor structure of the questionnaire. The resulting factors were compared with the factors generated at the design phase and in expert panel consultation to determine TBMAS's construct validity.

#### Content validity

it was assessed by Pearson correlation between TBMAS scores and the sum scores for items within each factor.

#### Convergent validity

it was assessed by Pearson correlation between CMG score and TBMAS scores as well as factor scores.

#### Receiver Operating Characteristics (ROC) analysis and TBMAS cut-off point in identifying non-adherents

The primary aim of TBMAS was to identify non-adherents and to explore specific barriers to adherence, therefore a clinically meaningful cut-off point of the TBMAS score that separates non-adherents from adherents is pivotal. This was done with ROC curve analysis of TBMAS score using CMG score as criteria.

#### Positive predictive value and sensitivity of TBMAS in identifying non-adherents

The positive predictive value (PPV) and sensitivity of TBMAS were calculated by examining the concordance and discordance between non-adherents identified by TBMAS with 113 as cut-off and CMG, using CMG as gold standard. PPV was calculated as the percentage of non-adherents identified by TBMAS that were confirmed by CMG, and sensitivity was calculated as the percentage of non-adherents identified by CMG that were also identified by TBMAS.

All analyses were performed using SPSS, version 12.0 (SPSS Inc.,Chicago, IL, USA).

## Results

Table 1 shows the characteristics of the 438 TB patients who completed TBMAS. There were 1 to 3 missing values across the 41 items for 12 patients, and the missing value of an item was inputted by the median of the non-missing item scores. The minimum, maxim, average and standard deviation of the TBMAS scores are 103, 179, 143.12 and 12.56, respectively. The average time to complete the questionnaire was 15∼20 minutes.

**Table 1:** Demographics of the participating patients (n = 438).

Demographic characteristics	Percentage (%)
Gender	
Male	68.16
Female	31.84
Age	
≤20	13.95
20∼40	40.90
40∼60	35.93
≥60	9.22
Education Level	
Junior high school or lower	33.10
Senior high school	40.28
College or higher	26.62
Marital Status	
Unmarried	36.24
Married	59.06
Divorce or Widowed	4.71

Pharmacy refill records for a 15-week period were available for all 438 patients, and the minimum, maxim, average and standard deviation of their CMG scores were 0, 0.55, 0.16 and 0.12, respectively. With CMG<0.2 as cut-off point as others have done [20], the CMG measurement identified 181(41.3%) of the 438 patients as non-adherents.

Seven of the 41 items on the initial TBMAS were removed for lacking discrimination power. Three items were removed because they were not statistically significantly associated with CMG score. The exploratory factor analysis performed on the remaining 31 items indicates that one item had substantial cross-loading on many factors, and therefore was removed, resulting in a 30-item TBMAS.

Catell's scree plot analysis indicated that the factor structure was best described by nine factors. Thus, a solution with nine factors was attempted using Varimax rotation, with cumulative variance accounting for by the nine-factor solution at 65%. Table 2 presents the nine factors, their corresponding items and factor loadings produced from the exploratory factor analysis. These nine factors correlate well with the nine factors conceptualized in the design phase, and the 30 items showed salient loadings on specific factors without substantial cross-loadings on the other factors, indicating good construct validity.

**Table 2:** TBMAS Factor loadings.

Item	Factor loadings								
	F1	F2	F3	F4	F5	F6	F7	F8	F9
**Factor I: communication with healthcare provider**									
I am satisfied with healthcare worker's attitude.	0.74	0.05	−0.07	0.04	0.08	0.13	0.08	0.09	0.21
Healthcare worker described TB to me clearly.	0.83	0.03	0.07	0.09	0.00	0.04	0.09	−0.01	0.09
Healthcare worker explained my condition to me clearly.	0.84	0.02	0.08	0.09	0.01	0.06	0.13	−0.03	0.04
Healthcare worker explained the method of taking medicine clearly.	0.78	0.10	0.10	0.10	0.05	0.09	0.02	0.00	0.17
Healthcare worker explained the side-effects of medicine clearly.	0.77	−0.01	0.12	0.18	0.00	−0.01	0.04	0.05	0.12
Healthcare worker led me to believe that my TB can be cured.	0.61	0.03	0.45	0.10	0.13	−0.01	0.07	0.04	−0.17
**Factor II: personal traits**									
I often keep my things neat and clean.	0.17	0.65	0.03	0.15	−0.07	0.16	0.14	0.07	−0.13
I am strict with myself to follow my plan.	0.00	0.75	0.06	0.14	0.10	0.05	0.17	0.13	0.08
I often seek the most effective way in doing things.	−0.03	0.71	0.11	0.27	0.07	−0.07	0.02	−0.03	0.14
I often set clear target.	0.07	0.82	0.11	0.06	0.15	0.00	0.06	0.09	−0.01
I am organized and systematic in approaching my target.	−0.03	0.43	0.17	0.23	0.21	0.14	0.01	0.15	0.32
**Factor III: confidence in curing TB**									
I am very confident to completely cure TB.	0.32	0.13	0.69	0.13	0.20	−0.11	0.07	0.08	−0.09
My treatment regimen is very simple.	−0.09	0.10	0.63	0.04	0.11	0.05	−0.03	−0.10	0.14
I am very confident in taking TB medicine regularly for 6 months.	0.21	0.08	0.77	0.08	0.02	0.11	0.12	0.19	0.00
I am very confident in tolerating side-effects.	0.10	0.07	0.67	0.23	−0.07	0.14	0.07	0.09	0.17
**Factor IV: social support**									
I am satisfied with the support between our family members.	0.08	0.06	0.30	0.63	0.08	0.13	0.05	0.06	0.24
My family members often remind me to take medicine.	0.10	0.17	0.13	0.77	−0.03	0.00	0.06	0.02	0.06
My friends often remind me to do things.	0.12	0.26	0.03	0.73	0.00	−0.01	0.09	−0.03	−0.10
People around me often give me necessary help.	0.28	0.17	0.07	0.63	0.03	0.08	0.11	−0.05	−0.01
**Factor V: mood disorders**									
I sometimes feel depressed.	0.06	−0.02	0.14	0.10	0.73	0.08	0.03	0.21	−0.01
When I do something wrong, I feel frustrated and want to give up.	0.02	0.10	0.02	−0.04	0.73	0.07	0.03	0.11	0.06
I sometimes feel helpless and want other people's help.	0.07	0.17	0.05	0.00	0.76	0.04	0.00	−0.01	0.10
**Factor VI: living habits**									
I sleep and wake up regularly every day.	0.07	0.08	0.06	0.09	0.07	0.88	0.01	0.04	0.00
I have meals regularly every day.	0.15	0.05	0.11	0.04	0.12	0.86	0.06	0.05	0.06
**Factor VII: active coping behavior**									
I actively pursued knowledge on TB when I knew I had been infected.	0.10	0.14	0.05	0.12	0.02	0.06	0.87	0.03	0.02
I often ask the doctor about my condition since I know I have been infected.	0.22	0.21	0.13	0.14	0.04	0.01	0.81	−0.03	−0.02
**Factor VIII: forgetfulness**									
I sometimes forgot to do important things I planned to do.	−0.04	0.06	0.10	−0.08	0.14	0.05	0.07	0.78	0.08
My memory is good.	0.12	0.19	0.06	0.07	0.14	0.04	−0.08	0.77	−0.02
**Factor IX: access to healthcare**									
It's convenient to refill my TB medicine.	0.23	0.05	0.05	0.01	0.14	0.06	0.10	0.01	0.77
The TB control institution I visit meets my need.	0.35	0.04	0.14	0.07	0.00	−0.02	−0.12	0.03	0.61

Cronbach's alpha was 0.87 for the entire TBMAS, and 0.88, 0.78, 0.73, 0.75, 0.67, 0.78, 0.77, 0.51 and 0.52 for the nine factors, respectively; test-retest reliability was 0.83; and split-half reliability was 0.85; all of which suggest TBMAS's robust reliability.

Table 3 presents TBMAS's content validity, supported by the statistically significant correlations between the total TBMAS scores and the nine factor scores, and TBMAS's convergent validity was supported by the statistically significant correlations between CMG scores and the total TBMAS scores as well as the nine individual factor scores.

**Table 3:** The correlation between TBMAS factor score and total TBMAS score and between TBMAS score and CMG score.

Sum Score	TBMAS Score		CMG Measurement	
	Pearson correlation coefficient	P-value	Pearson correlation coefficient	P-value
Entire Scale	1	—	0.6051	<0.0001
Factor I	0.6994	<0.0001	0.3800	<0.0001
Factor II	0.6610	<0.0001	0.3385	<0.0001
Factor III	0.6534	<0.0001	0.5308	<0.0001
Factor IV	0.6579	<0.0001	0.4109	<0.0001
Factor V	0.4729	<0.0001	0.2957	<0.0001
Factor VI	0.4291	<0.0001	0.2774	<0.0001
Factor VII	0.5111	<0.0001	0.2771	<0.0001
Factor VIII	0.4719	<0.0001	0.2807	<0.0001
Factor IX	0.3810	<0.0001	0.2442	<0.0001

**Figure 1:**
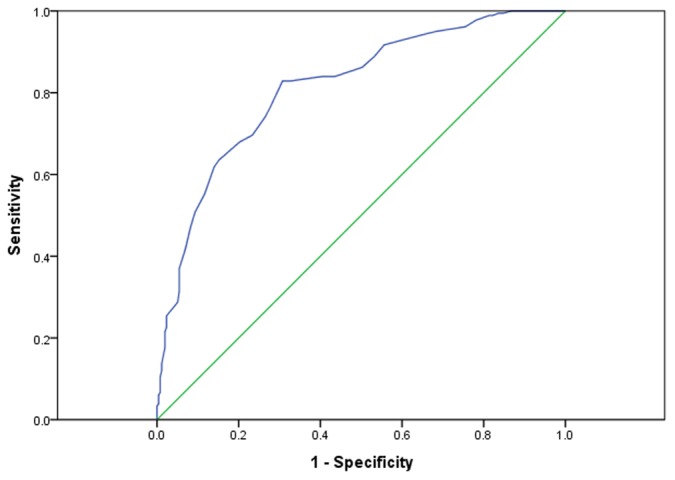
ROC curve for TBMAS total score using CMG score as criteria.

Figure 1 presents the ROC curve computed for total score using CMG score as criteria. The area under the ROC curve was 0.82 (95% CI: 0.77–0.86), indicating TBMAS's high predictive value. The cut-off point for labeling non-adherent was identified where the total TBMAS score is at 113. With this cut-off point, 229 (52.3%) of the 438 participating patients were considered non-adherents.

Table 4 shows the concordance and discordance between TBMAS and CMG scores in identifying non-adherents, when TBMAS score below 113 are considered non-adherents. Using CMG as gold standard, TBMAS has a PPV of 65.5% and sensitivity of 82.9% in identifying non-adherents.

**Table 4:** Concordance between of TBMAS and CMG in identifying non-adherents.

TBMAS	CMG		Total
	Non-adherent	Adherent	
Non-adherent	150	79	229
Adherent	31	178	209
Total	181	257	438

## Discussion

In this study, we produced a 30-item TBMAS and showed that TBMAS is fairly consistent, reliable and valid. We demonstrated that, at the cut-off point of 113, TBMAS has a positive predictive value of 65.5% and sensitivity of 82.9% in identifying non-adherents among TB patients, suggesting that TBMAS can be a reasonably valid screening tool for TB treatment professionals. Besides the series of statistics presented above, TBMAS determined that 52.3% of the 438 participants were non-adherents and the CMG identified 41.3% as non-adherents, which are consistent with the overall adherence rates reported in the literature. For examples, the WHO “World Medicines Situation, 2011” pointed out that patient adherence to treatment was about 50% worldwide and lower in developing and transitional countries [21]; and a study in Hubei province observed that 46% of TB patients missed at least 10% of their doses [22]. This consistency further supports the validity of TBMAS.

TBMAS has many advantages over the existing instruments or approaches to assess non-adherence in TB patients. By going beyond pill counts or pharmacy record review or simply asking whether a patient is taking medicine as directed, TBMAS delves into the possible reasons for non-adherence, and suggests targeted interventions to improve adherence. For example, patients with forgetfulness can be given special containers or reminders. Patients who lack confidence in curing their TB or lack active coping ability can be given further counseling and education [14]. Doctors can act more actively and proactively in engaging family members for patients who lack social support. By analyzing patients' answers to TBMAS, healthcare providers will realize whether they have adequate communication with their patients, and, if not, they will know which aspects of the communication is problematic and what corrective actions need to be taken.

Several limitations of this study need to be noted. First, we used CMG as gold standard to validate TBMAS, even though we recognize that CMG based on pharmacy record review has its own limitations in measuring medication adherence [19], [20]. Consequently, our validation statistics may be biased one way or another. Second, pharmacy refill records offer an objective observation on whether patients get their refills on time, but there is no guarantee that obtained medicine are actually taken [19], therefore, CMG may over-estimate medication adherence and consequently bias our estimates of TBMAS's validity. Third, the validation study was conducted in one urban area, and may not be representative of TB patients from rural areas or other socio-economically different areas. These limitations could be addressed in the future when TBMAS is used in the field and evaluated in different patient populations with different validity criteria.

TB remains one of the biggest public health threats in China and poses many great challenges [23]. Under WHO's leadership, China has faithfully implemented DOTS and achieved great success in TB control. However, DOTS has fallen short in many ways, with reported proportion of patients who took their medicine under the surveillance of doctors varying from 0% to 70% in different counties [6], [7], [24]. In many areas, DOTS is completely absent, where patients visit TB clinics monthly and get anti-tuberculosis drugs for a whole month without any supervision [25]. As DOTS's limitations, especially DOTS's total reliance on medical professionals in medication adherence, are increasingly recognized, China has developed many alternative strategies that place more adherence responsibility on individual TB patients. For example, in the city of Wuhan, three management strategies have been implemented concurrently, DOTs, DOT in the treatment initiation stage and patient self-supervised chemotherapy, with patient self-supervision accounting for about 5% of Wuhan's total TB cases [26]. Under these circumstances, TBMAS can be a powerful tool for TB medical professionals to screen potential non-adherents and proactively intervene in order to ensure and improve medication adherence.
